# Diversity of *Rhizophydiales* (*Chytridiomycota*) in Thailand: unveiling the hidden gems of the Kingdom

**DOI:** 10.1186/s43008-024-00144-x

**Published:** 2024-06-27

**Authors:** Vedprakash G. Hurdeal, Joyce E. Longcore, E. B. Gareth Jones, Kevin D. Hyde, Eleni Gentekaki

**Affiliations:** 1 School of Science, Mae Fah Luang University, Chiang Rai, 57100 Thailand; 2 Center of Excellence in Fungal Research, Mae Fah Luang University, Chiang Rai, 57100 Thailand; 3 School of Biology and Ecology, University of Maine, Orono, ME 04469-5722 USA; 4 Department of Botany and Microbiology, College of Science, King Saud University, P.O. Box 2455, Riyadh, 11451 Saudi Arabia; 5 Department of Veterinary Medicine, University of Nicosia School of Veterinary Medicine, Nicosia, 2414 Cyprus

**Keywords:** 8 new species, Fungal diversity, Novel species, Phylogeny, *Rhizophydiales*, Species delimitation, Zoosporic fungi

## Abstract

Chytrids, often overshadowed by their other fungal counterparts, take center stage as we unravel the mysteries surrounding new species within *Rhizophydiales* and explore their unique characteristics. In the broader spectrum of chytrids, their significance lies not only in their roles as decomposers but also as key players in nutrient cycling within aquatic ecosystems as parasites and saprobes. Baited soil and aquatic samples collected from various provinces of Thailand, yielded new species of the *Rhizophydiales* (*Chytridiomycota*), some of which expanded previously single species genera. Our investigation incorporated a combination of morphological and phylogenetic approaches, enabling us to identify these isolates as distinct taxa. The novel isolates possess distinguishing features, such as variations in size and shape of the sporangium and zoospores, that somewhat differentiate them from described taxa. To confirm the novelty of the species, we employed robust phylogenetic analyses using maximum likelihood and bayesian methods. The results provided strong support for the presence of eight distinct lineages within the *Rhizophydiales*, representing our newly discovered species. Furthermore, we employed Poisson Tree Processes to infer putative species boundaries and supplement evidence for the establishment of our new *Rhizophydiales* species. By meticulously exploring their morphological characteristics and genetic makeup, we expand the known catalogue of fungal diversity by describing *Alphamyces thailandicus*, *Angulomyces ubonensis, Gorgonomyces aquaticus, G. chiangraiensis*, *G. limnicus*, *Pateramyces pingflumenensis*, *Terramyces aquatica,* and *T. flumenensis* and also provide valuable insights into the intricacies of this order. This newfound knowledge not only enriches our understanding of *Rhizophydiales* but also contributes significantly to the broader field of mycology, addressing a critical gap in the documentation of fungal species. The identification and characterization of these eight novel species mark a noteworthy stride towards a more comprehensive comprehension of fungal ecosystems and their vital role.

## Introduction

The kingdom *Fungi* comprises various distinct lineages namely, *Dikarya*, zygosporic and zoosporic taxa (Voigt et al. 2021; Wijayawardene et al. 2022). Among all the known fungal groups, zoosporic lineages are least studied (Hurdeal et al. 2020). Of these, the *Chytridiomycota*, commonly referred to as chytrids, is the most abundant and studied lineage (Letcher et al. 2015; Seto and Degawa 2018a; Longcore and Simmons 2020; Hurdeal et al. 2020; Voigt et al. 2021). Chytrids are predominantly found in aquatic systems. With the emergence of next generation sequencing data, we have evidence that chytrids represent a significant portion of the overall fungal community, however they are also isolated from terrestrial habitats (Panzer et al. 2015; Hassett et al. 2020; Longcore and Simmons 2020; Blaalid and Khomich 2021; Van den Wyngaert et al. 2022). Saprobic chytrids are detected on baits such as pollen grains, onion skin, and chitin among others (Longcore and Simmons 2020). Parasitic chytrids infect hosts ranging from green algae to amphibians in various aquatic niches (Fisher and Garner 2020; Seto et al. 2020a).

Identification of chytrids by light microscopy is often difficult because many species have similar morphology; consequently, species descriptions are primarily based on molecular phylogenetic analyses using the internal transcribed spacer and large subunit ribosomal ribonucleic acid (ITS-LSU) genetic markers (James et al. 2000) and previously ultrastructural details of the zoospores (Letcher et al. 2006). Recently, Hurdeal et al. (2023) proposed a polyphasic approach employing phylogeny, Poisson tree processes, and genetic distance analysis of the ITS to describe novel species (Schoch et al. 2012). This approach was proposed as morphology alone is insufficient to identify new taxa at the species level.

The systematics of *Rhizophydiales* has been continuously updated over the last few years. Molecular phylogeny and zoospore ultrastructure studies have enabled the elevation of the problematic genus *Rhizophydium sensu lato* to the order *Rhizophydiales*, within which Letcher, Powell and others, established new genera and neo-typed historical species (Letcher et al. 2006, 2008, 2012). The historical chytrid literature has numerous morphologically described *Rhizophydium* species that are currently taxonomically unresolved because attempts to match new isolates to descriptions based on organisms not in axenic culture can be erroneous and therefore is problematic (Hurdeal et al. 2023). Intricacies, such as various species with different lifestyles, and the number of historical species makes this order particularly interesting to study.

Thailand is a tropical country with an immense diversity of flora, fauna, and funga with a significant potential for organismal discovery. In the last decade, descriptions of new fungal species from Thailand have contributed significantly to fungal taxonomy. Though most studies were and are still, primarily focused on dikaryan fungi, attempts to study basal lineages in the country are emerging (Hurdeal et al. 2021a, b, 2022, 2023). To discover and explore zoosporic fungi, we collected and baited soil and water samples from different locations. We implemented a polyphasic approach using phylogeny, Poisson tree processes (PTP) and morphology to classify recovered *Rhizophydiales* isolates resulting in new species in *Alphamyces*, *Angulomyces*, *Gorgonomyces*, *Pateramyces, and Terramyces*. The new species and their phylogenetic relationships increase our knowledge of the *Rhizophydiales* in Thailand and also reveal the relationship of the new species to rhizophydialean species from other parts of the world.

## Materials and methods

### Sample collection, and isolation

We collected soil and water samples from Chiang Rai, Chiang Mai and Ubon Ratchathani, Thailand. Water samples, along with some sediments, were collected from various lakes and rivers in sterile 50 mL centrifuge tubes. Soil samples were collected from the rhizosphere of shrubs and trees. Surface organic debris was manually removed and a sterile shovel or spoon was used to dig the soil, which was stored in Ziploc bags or centrifuge tubes.

For isolation, all samples were baited with pine pollen. Water samples were poured into 90 mm plastic Petri dishes, and pollen added. 1 g of soil was weighed and transferred to a sterile 90 mm Petri plate. The soil was then flooded with sterilized water and pollen added. All inoculated plates were kept at 20 °C and monitored daily under 100–400X magnification (Nikon Eclipse Ni). Once infected pollen grains were observed, a single sporangium was transferred with a drawn micropipette or a needle to PmTG agar medium supplemented with streptomycin sulfate (350 mg/L) and penicillin G (200 mg/L) (Barr 1986). Morphological characters of the fungi were observed with a Nikon Eclipse Ni compound microscope (100–600X) under DIC and images taken with a Nikon DS-RI2 digital camera. Isolates were preserved in 15% glycerol and following the protocol recommended by Collection of Zoosporic Eufungi at the University of Michigan (CZEUM) for long-term preservation (https://czeum.herb.lsa.umich.edu/). Ex-type living cultures have been deposited in the Mae Fah Luang culture collection (MFLUCC), Chiang Rai, Thailand. Photoplates and species descriptions have been deposited in MycoBank.

### DNA extraction, genetic marker amplification, and sequencing

Genomic DNA was extracted from mature cultures using G-spin™ Total DNA Extraction Kit (Intron Biotechnology, South Korea) following the manufacturer’s instructions. Amplicons of partial fragments of ITS1-5.8S-ITS2, and LSU were generated with polymerase chain reaction (PCR). The primers used were ITS4/ITS5 and LROR/LR5 (Vilgalys and Hester 1990; White et al. 1990). PCR conditions were initial denaturation at 94 °C for 5 min, followed by 30 cycles of denaturation at 94 °C of 1 min, annealing at 52 °C for 45 s, elongation at 72 °C for 90 s and final elongation at 72 °C for 7 min.

PCR products were purified with the MEGAquick spin plus fragment DNA purification kit (Intron Biotechnology, South Korea). Sequencing was performed with an Applied Biosystems 3130XL DNA analyzer (Bionics, South Korea).

### Phylogenetic analysis and poisson tree processes

Raw DNA sequence data were edited and assembled into contigs using SeqMan Version 7.1.0. The newly generated sequences were used as queries to perform blast searches against the nucleotide database (nr) in GenBank to check for possible contamination and to assist with taxon sampling (Altschul et al. 1990). The dataset for *Rhizophydiales* followed Hurdeal et al. (2023) and was updated to include newly introduced taxa (see Table 1). Taxon sampling within identified genera where new species are being introduced spanned the genetic diversity currently available. Only ITS and LSU were used as genetic markers, as they are the most broadly available. Although, the small subunit ribosomal RNA (SSU) marker is also quite well represented for chytrids, for this order, the number of SSU sequence data available is low (< 40% of overall taxa used in this dataset). Hence the marker was not included in our analysis. Data for Rhizophydiales taxa were extracted from GenBank and CZEUM. Datasets for each genetic marker were built and aligned using MAFFT on the online webserver (https://mafft.cbrc.jp/alignment/server/) and trimmed with TrimAl Version 1.2 (Katoh and Toh 2008; Capella-Gutiérrez et al. 2012). The two individual datasets were concatenated into a single matrix, which was used for the final phylogenetic analysis.

**Table 1:** Data used for phylogenetic analysis of *Rhizophydiales* in this study, their corresponding GenBank accession numbers, source and habitats. Type (T), epitype (ET), ex-type (EX), and neotype (NT) species are denoted by superscripts to species names. Sequences derived in this study are shown in bold

Species name	Strain	Accession Number		Source	Habitat/substrate
		**ITS**	**LSU**		
*Alphamyces chaetifer*	ARG-110	JF809849	JF809854	Entre Ríos, Argentina	Aquatic
*Alphamyces chaetifer*	MP-047	JF809851	JF809856	Alabama, USA	Aquatic
*Alphamyces chaetifer*^ET^	ARG-025	NR_119646	NG_060383	Corrientes, Argentina	Stream/pollen
***Alphamyces thailandicus***^**T**^	**MFLUCC 23–0069**	**OR051769**	**OR051780**	**Ubon Ratchathani Province, Thailand**	**Water/sandy sediment/pollen**
*Angulomyces argentinensis*^EX^	ARG-008	NR_119644	NG_042447	Buenos Aires, Argentina	Stream/pollen
*Angulomyces argentinensis*	ARG-070	EF585667	EF585627	Capital Federal, Argentina	Aquatic/pollen
*Angulomyces solicola*	MFLUCC 22–0100	ON899833	ON892504	Chiang Mai Province, Thailand	Soil/pollen
*Angulomyces solicola*^T^	MFLUCC 22–0101	ON899834	ON892505	Chiang Rai Province, Thailand	Soil/pollen
***Angulomyces ubonensis***	**MFLUCC 23–0297**	**OR051767**	**OR051778**	**Ubon Ratchathani Province, Thailand**	**Muddy river/pollen**
***Angulomyces ubonensis***^**T**^	**MFLUCC 23–0072**	**OR051768**	**OR051779**	**Ubon Ratchathani Province, Thailand**	**Muddy river/pollen**
*Aquamyces chlorogonii*^ET^	ARG-018	EF585643	EF585603	Buenos Aires, Argentina	Semi-permanent roadside pond /pollen
*Aquamyces chlorogonii*	ARG- 020	EF585644	EF585604	Buenos Aires, Argentina	Aquatic/pollen
*Aquamyces chlorogonii*	JEL-317	AY997081	DQ273779	Maine, USA	Soil/*Haematococcus*
*Betamyces americaemeridionalis*	ARG-063	EF585664	EF585624	Buenos Aires, Argentina	Vegetated roadside pond /pollen
*Boothiomyces macroporosum*	CBS-122107	MH863177	MH874723	New South Wales, Australia	Soil, pine pollen
*Boothiomyces macroporosum*^ET^	PL-AUS-021	NR_119591	AY439040	New South Wales, Australia	Soil, pine pollen
*Boothiomyces macroporosum*	WJD128	MT731002	KC691381	Alabama, USA	Aquatic/pollen
*Boothiomyces* sp.	JEL055/Barr 089	DQ485611	DQ485547	British Columbia, Canada	Halophytic soil/pollen
*Boothiomyces* sp.	JEL348	DQ485624	DQ485558	Maine, USA	Aquatic/pollen
*Collimyces mutans*	KS100	LC274663	LC274662	Chiba, Japan	Aquatic/*Microglena coccifera*
*Coralloidiomyces digitatus*	UACCC-PL-163L	NR_119652	NG_042452	Chubut Province, Argentina	Soil/pollen
*Dinomyces arenysensis*	P236	KJ027546	KJ027545	Arenys de Mar harbour, Mediterranean Sea, Spain	Aquatic (M)/ *Alexandrium minutum*
*Dinomyces arenysensis*	P237	KJ027548	KJ027547	Arenys de Mar harbour, Mediterranean Sea, Spain	Aquatic (Marine)/ *Alexandrium minutum*
*Gammamyces ourimbahensis*	PL-116	DQ485670	DQ485579	New South Wales, Australia	Soil/pollen
*Globomyces pollinis*	ARG-069	EF585666	EF585626	Capital Federal, Argentina	Aquatic/pollen
*Globomyces pollinis*^ET^	ARG-068	NR_119649	NG_042451	Capital Federal, Argentina	Aquatic (lake)/pollen
*Globomyces pollinis*	Barr-003	DQ485596	DQ485532	Michigan, USA	Aquatic/*Pediastrum*
*Gorgonomyces thailandicus*	MFLUCC 22–0098	ON899835	ON892506	Chiang Rai Province, Thailand	Aquatic/pollen
*Gorgonomyces thailandicus*^T^	MFLUCC 22–0099	ON899836	ON892507	Chiang Rai Province, Thailand	Aquatic/pollen
*Gorgonomyces* sp.	ARG-029	EF585650	EF585610	Corrientes, Argentina	Aquatic/pollen
*Gorgonomyces* sp.	ARG-036	EF585654	EF585614	Corrientes, Argentina	Marsh/pollen
*Gorgonomyces* sp.	BARR100	DQ485599	DQ485535	Quebec, Canada	Aquatic/Cladophora
*Gorgonomyces haynaldii*^ET^	BAFC-ARG-026	NR_119647	NG_042448	Corrientes, Argentina	Aquatic/pollen
*Gorgonomyces* sp.	Arg-024	EF585645	EF585605	Corrientes, Argentina	Aquatic/pollen
*Gorgonomyces* sp.	ARG-119	MT730618	MT730618	Argentina	-
*Gorgonomyces* sp.	ARG-120	MT730619	MT730619	Argentina	-
*Gorgonomyces* sp.	ARG-125	MT730622	MT730622	Argentina	-
*Gorgonomyces* sp.	JEL0862	MT730856	MT730856	Maine, USA	Mud Pond, pollen
*Gorgonomyces* sp.	JEL0887	MT730869	MT730869	Maine, USA	Mud Pond, pollen
*Gorgonomyces* sp.	JEL0923	MT730896	MT730896	Texas, USA	Aquatic/pollen
*Gorgonomyces* sp.	JEL0930	MT730897	MT730897	Maine, USA	Aquatic/pollen
*Gorgonomyces* sp.	JEL0957	MT730914	MT730914	Maine, USA	Aquatic/pollen
*Gorgonomyces* sp.	JEL0964	MT730919	MT730919	Maine, USA	Aquatic/Chitin
*Gorgonomyces* sp.	JEL0965	MT730920	MT730920	Maine, USA	Aquatic/Chitin
*Gorgonomyces* sp.	JEL151	AY997080	DQ273774	Maine, USA	Aquatic/*Lyngbya*
*Gorgonomyces* sp.	MP57	MT730942	MT730942	Madison County, Alabama, USA	Aquatic
***Gorgonomyces aquaticus***^**T**^	**MFLUCC 23–0296**	**OR051771**	**PP051500**	**Chiang Rai Province, Thailand**	**Aquatic/pollen**
*Gorgonomyces* sp.	WJD130	-	KC691383	Alabama, USA	Aquatic/bait
***Gorgonomyces limnicus***^**T**^	**MFLUCC 23–0066**	**OR051770**	**OR051781**	**Chiang Rai Province, Thailand**	**Aquatic/pollen**
*Gorgonomyces limnicus*	UM1559	MT730975	MT730975	Michigan, USA	Aquatic/pollen
***Gorgonomyces chiangraiensis***^***T***^	**MFLUCC 23–0070**	**OR051772**	**OR051782**	**Chiang Rai Province, Thailand**	**Aquatic/pollen**
***Gorgonomyces chiangraiensis***	**MFLUCC 23–1307**	**OR051773**	**OR051783**	**Chiang Rai Province, Thailand**	**Aquatic/pollen**
*Halomyces littoreus*	Barr-263	DQ485604	DQ485540	Virginia, USA	Aquatic/*Bryopsis plumosa*
*Kappamyces betamyces*	Barr-316	DQ485606	DQ485542	New Brunswick, Canada	Salt marsh/pollen
*Kappamyces laurelensis*	AFTOL-ID-690	DQ536494	DQ273824	Georgia, USA	Soil/pollen
*Kappamyces laurelensis*	CBS-122106	MH863176	MH874722	Georgia, USA	Soil/pollen
*Kappamyces laurelensis*^EX^	PL098	NR_119595	NG_060251	Georgia, USA	Soil/pollen
*Kappamyces* sp.	JEL356	DQ485625	DQ485559	California, USA	Soil/pollen
*Kappamyces* sp.	PL117	EF585670	EF585630	Virginia, USA	Soil/pollen
*Kappamyces* sp.	PL118	DQ485671	DQ485580	Virginia, USA	Soil/pollen
*Operculomyces laminatus*^T^	JEL-223	NR_119590	NG_042440	Maine, USA	Soil/snake skin keratin
*Paludomyces mangrovei*^T^	ATCC-26191	NR_138404	NG_059549	Sao Paulo, Brazil	Mangrove swamp sediment/pollen and cattle hair
*Paranamyces uniporus*	JEL-695	KP723824	KP723818	Maine, USA	Soil/pollen
*Paranamyces uniporus*^T^	PL157	DQ485685	DQ485594	Buenos Aires, Argentina	Estuarine mud flat/pollen
*Paranamyces uniporus*	WJD-158	KP723827	KP723820	Alabama, USA	Soil/pollen
*Paranamyces uniporus*	WJD-193	KP723828	KP723821	Ohio, USA	Tamarack bog/keratin
*Pateramyces corrientinensis*	ARG-031	EF585651	EF585611	Capital Federal, Argentina	Aquatic/pollen
*Pateramyces corrientinensis*^EX^	ARG-046	NR_111261	NG_042449	Corrientes, Argentina	Aquatic (lake)/pollen
***Pateramyces pingflumenensis***^**T**^	**MFLUCC 23–0068**	**OR051766**	**OR051777**	**Chiang Mai Province, Thailand**	**River water/pollen**
*Polyrhizophydium stewartii*^EX^	JEL0888	MT730870	MT730870	Maine, USA	Aquatic/*Eriocaulon aquaticum*
*Polyrhizophydium stewartii*	JEL0932	MT730899	MT730899	Maine, USA	Aquatic/ *Eriocaulon aquaticum*
*Protrudomyces* sp.	JEL-134	DQ485612	DQ485548	Maine, USA	Aquatic/*Achlya*
*Protrudomyces lateralis*^EX^	ARG-071	NR_119650	NG_060073	Capital Federal, Argentina	Aquatic (lake)/pollen
*Protrudomyces lateralis*	Barr-004	DQ485597	DQ485533	Ontario, Canada	Aquatic/*Ulothrix*
*Rhizophlyctis rosea*	AFTOL-ID-43	AY997078	DQ273787	Georgia, USA	Soil
*Rhizophlyctis rosea*	PL132	EU379237	EU379194	Windermere, England	Soil
*Rhizophydiales* sp.	ARG-033	EF585652	EF585612	Capital Federal, Argentina	Aquatic/pollen
*Rhizophydium brooksianum*	AFTOL-ID-22	-	DQ273770	Maine, USA	Soil/pollen
*Rhizophydium brooksianum*^EX^	JEL-136	NR_119550	NG_060069	Maine, USA	Soil/pollen
*Rhizophydium echinocystoides*	B8	-	MH933969	Michigan, USA	Bog water/
*Rhizophydium globosum*	CBS-120403	MH863084	MH874643	Maine, USA	-
*Rhizophydium globosum*	JEL-222	DQ485616	DQ485551	Maine, USA	Soil/pollen
*Rhizophydium jobii*	OAS2	MN787065	MN759467	Salalah, Oman	Benthic detritus/pine pollen
*Rhizophydium jobii*^T^	OAS6	MN787067	MN759470	Salalah, Oman	Benthic detritus/pine pollen
*Rhizophydium koreanum*^T^	CNUFC-17CPW1-1	-	MH298649	Gwangju, South Korea	Pond water/
*Rhizophydium koreanum*	CNUFC-17CPW1-2	-	MH298650	Gwangju, South Korea	Pond water/
*Rhizophydium* sp.	ARG-013	EF585638	EF585598	Buenos Aires, Argentina	Aquatic/pollen
*Rhizophydium* sp.	ARG-014	EF585639	EF585599	Buenos Aires, Argentina	Aquatic/pollen
*Rhizophydium* sp.	ARG-035	EF585653	EF585613	Capital Federal, Argentina	Marsh/pollen
*Rhizophydium* sp.	BR1	AY349121	AY439057	-	-
*Rhizophydium* sp.	JEL292	DQ485620	DQ485554	Maine, USA	Aquatic/pollen
*Rhizophydium* sp.	JEL316	DQ536497	DQ273835	Maine, USA	Aquatic/pollen
*Rhizophydium* sp.	LL6	AY349122	AY439059	-	-
*Rhizophydium* sp.	MP050	-	KC691337	Alabama, USA	Aquatic
*Rhizophydium* sp.	PL-AUS-Ad014	DQ485647	DQ485570	New South Wales, Australia	-
*Rhizophydium* sp.	PL149A	DQ485682	DQ485591	Texas, USA	Soil/pollen
*Skeletonema parasitoid* ^T^	SkChyt5	MH643793	MH643793	Oban, UK	Aquatic/ *Skeletonema* sp.
*Spizellomyces punctatus*	ATCC-48900	NR_111189	NG_027618	Papua New Guinea	Soil/pollen
*Staurastromyces oculus*	STAU-CHY2	KY555735	KY555731	Oberhavel, Germany	Aquatic/ *Staurastrum* sp.
*Staurastromyces oculus*^T^	STAU-CHY3	KY350146	KY350145	Oberhavel, Germany	Aquatic/ *Staurastrum* sp.
***Terramyces aquatica***^**T**^	**MFLUCC 23–0298**	**OR051774**	**OR051784**	**Chiang Rai Province, Thailand**	**Lake water/pollen**
*Terramyces aquatica*	ARG-040	EF585656	EF585616	Corrientes, Argentina	Interface/pollen
*Terramyces chiangraiensis*^T^	MFLUCC 22–0102	ON899837	ON892508	Chiang Rai Province, Thailand	Forest soil/pollen
*Terramyces chiangraiensis*	MFLUCC 22–0103	ON899838	ON892509	Chiang Rai Province, Thailand	Forest soil/pollen
***Terramyces flumenensis***^**T**^	**MFLUCC 23–0067**	**OR051776**	**OR051786**	**Ubon Ratchathani Province, Thailand**	**Muddy river water/pollen**
***Terramyces flumenensis***	**MFLUCC 23–0071**	**OR051775**	**OR051785**	**Chiang Rai Province, Thailand**	**Lake water/pollen**
*Terramyces* sp.	PLAUS18	MT730963	AY439051	New South Wales, Australia	Soil/pollen
*Terramyces* sp.	JEL0393	DQ485627	DQ485561	New Zealand	Soil/pollen
*Terramyces* sp.	JEL0395	DQ485628	DQ485562	New Zealand	Soil/pollen
*Terramyces sphaerotheca*	JEL0302	DQ485623	DQ485557	Maine, USA	Soil/pollen
*Terramyces subangulosum*^ET^	PL-003	NR_119592	AY439041	Virginia, USA	Soil/pollen
*Terramyces subangulosum*	PL-122	DQ485673	DQ485582	Virginia, USA	Soil/pollen
*Uebelmesseromyces harderi*	AFTOL-ID-31	AY997077	DQ273775	-	-
*Uebelmesseromyces harderi*	ATCC-24053	DQ485595	AY349087	British Columbia, Canada	Intertidal soil/pine pollen
*Ulkenomyces aestuarii* ^NT^	ATCC-26190/Barr-303	DQ485605	DQ485541	Bremerhaven, Germany	Submersed estuary mud/ pine pollen
*Ulkenomyces aestuarii*	PL-137	DQ485676	DQ485585	Northern Cape, South Africa	Soil/ pollen
*Ulkenomyces aestuarii*	PL-190	KP723825	KP723819	British Columbia, Canada	Mud sample/keratin
*Urceomyces sphaerocarpus*^ET^	ARG-048	NR_119648	NG_042450	Corrientes, Argentina	Small lake (marsh)/pollen
*Urceomyces sphaerocarpus*	ARG-038	EF585655	EF585615	Corrientes, Argentina	Aquatic/pollen

The IQ-TREE was computed on the webserver https://iqtree.cibiv.univie.ac.at/ using the default parameters (Nguyen et al. 2015). Branch support was estimated from 1000 ultrafast bootstrap replicates. The analysis evaluated the best substitution model using Model Finder, which is embedded automatically in the analysis. The best model for *Rhizophydiales* was GTR + I + Γ for ITS and TIM3 + I + Γ for LSU. Maximum likelihood (ML) phylogeny using RAxML-NG Version 1.0.1 was inferred on the online CIPRES Portal with bootstrap support from 1000 pseudoreplicates (Miller et al. 2010; Kozlov et al. 2019). The combined data ML analysis was performed by partitioning the matrix according to the genetic markers (ITS and LSU) used and the best-suited models for each marker. The nucleotide substitution model for each genetic marker was evaluated using jModelTest2 on XSEDE in the online CIPRES Portal (https://www.phylo.org/portal2) (Miller et al. 2010; Darriba et al. 2012). The best model under the AIC criterion was TPM2uf + I + Γ for ITS and GTR + I + Γ for LSU. Maximum likelihood phylogenetic analysis was performed for each dataset separately and the concatenated matrix. Bayesian inference (BI) analysis was performed using MrBayes Version 3.2.7a (Huelsenbeck and Ronquist 2001). Four simultaneous chains were run for 2 000 000 generations with a sampling frequency of 100. 25% of the trees were discarded as burn-in. Convergence was declared when the standard deviation of split frequencies was less than 0.01. The final concatenated matrix and ML tree was deposited to Figshare (10.6084/m9.figshare.24910779). Newly generated sequences were deposited into GenBank (www.ncbi.nlm.nih.gov/genbank/).

To infer species boundaries, the coalescent-based Poisson tree processes (PTP) model was used (Zhang et al. 2013). PTP uses branch lengths (number of substitutions), which was extracted from the phylogenetic tree and added to infer branching events. The model assumes that the number of substitutions between species is significantly higher than within a species (Zhang et al. 2013). The analysis was performed on the online platform https://species.h-its.org/ptp/ and consisted of 100 000 Markov chain Monte Carlo (MCMC) generations, a thinning set to 100 and burn-in at 10%. The dataset comprised two genetic markers, ITS and LSU. Genus level ML (IQ-TREE) phylogenetic trees were computed. All analyses contained an outgroup, but a command to automatically remove distantly related outgroups to improve the delimitation results was implemented. Genetic distances (pairwise nucleotide substitution) were measured using the Kimura 2-parameter substitution model as implemented in MEGA-X with gamma distribution and pairwise deletion options. For the calculation, the trimmed alignments of ITS were used.

## Results

**Fig. 1:**
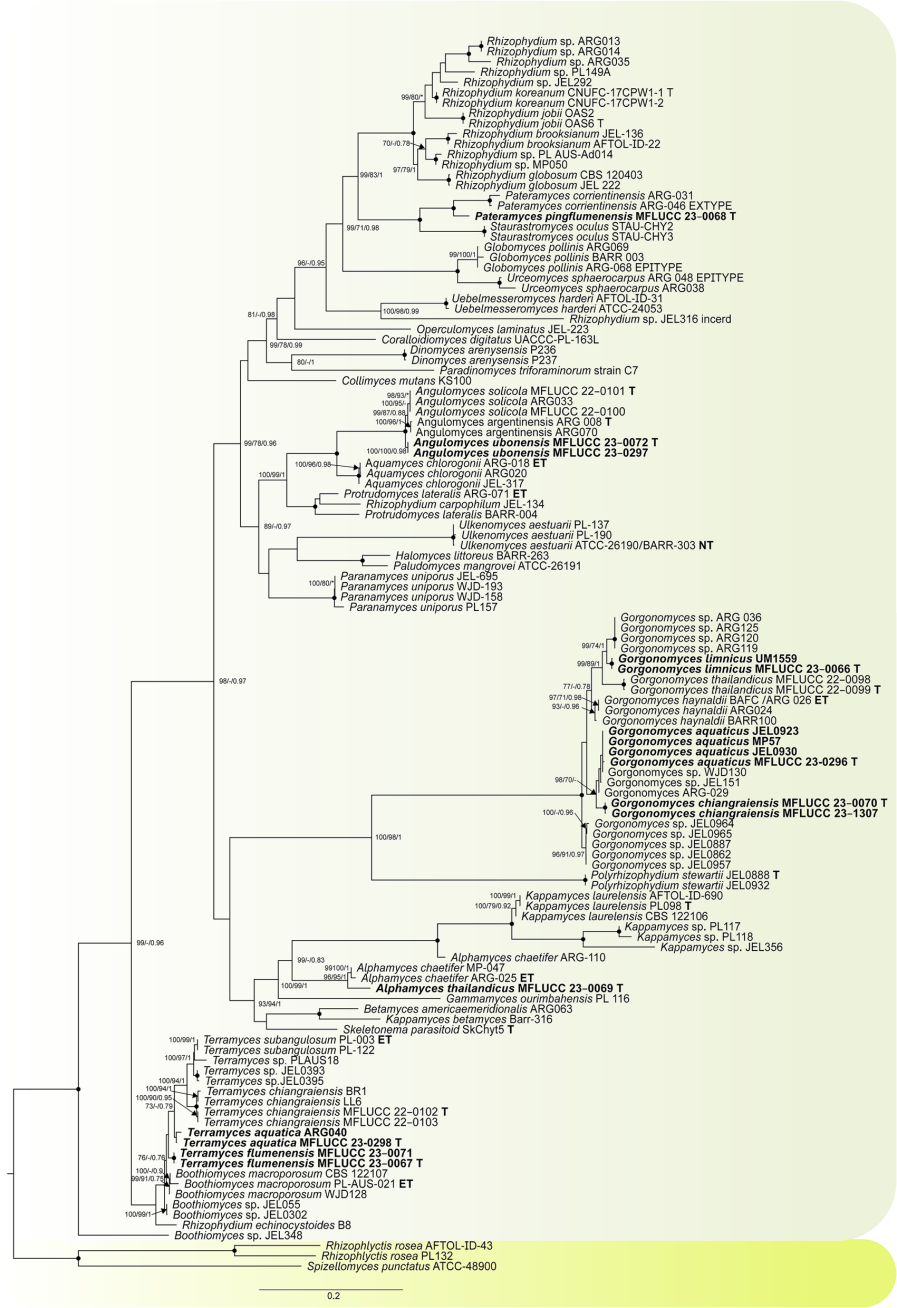
Maximum likelihood phylogram (IQ-tree) inferred from 123 taxa and 1645 characters based on a combined matrix consisting of ITS and LSU. Bootstrap support (RAxML/ IQ) (≥ 70%) and Bayesian posterior probabilities (≥ 0.70) are indicated above the branches or near the nodes in this order. Round nodes indicate maximum statistical support (100/100/1). The tree is artificially rooted using *Rhizophlyctis rosea* (AFTOL-ID-43), *R. rosea* (PL132), and *Spizellomyces punctatus* (ATCC-48900). The new species are in bold. (-) represent bootstrap support lower than 70% or for PP lower than 0.80. (*) indicates unrecovered branching

We found chytrids on all of our pollen-baited samples. The overall morphology of the isolates was reminiscent to the members of the Rhizophydiales but we noticed slight differences in the culture morphology. Chytrids from the gross cultures grew readily on PmTG and mPmTG agar plates resulting in 11 isolates, which represent eight new species from both terrestrial and aquatic habitats (Table 1). We constructed a *Rhizophydiales* phylogenetic tree (Fig. 1), with sequence data of 123 taxa from GenBank. In the final trimmed alignment, ITS comprised 713 and LSU 932 sites and the likelihood of the best scoring tree of the ML analysis was -31204.907.

The topology of the phylogenetic trees from the RAxML, Bayesian, and IQ-TREE analyses was congruent. The placement of the novel taxa remained unchanged in both single and concatenated matrix phylogenetic analyses. In all phylogenetic trees, the new strains formed clades distinct from the reference specimens. The position of nearly all new taxa was stable with high or maximum bootstrap support and posterior probability.

The *Angulomyces* isolates grouped together but consistently separated from the two known species: *A. argentinensis* and *A. solicola* (Fig. 1). Phylogenetic analyses also confirmed the placement of MFLUCC 23–0298, MFLUCC 23–0067 and MFLUCC 23–0071 within *Terramyces* and MFLUCC 23–0296, MFLUCC 23–0066 and MFLUCC 23–0070 within *Gorgonomyces*. The isolate MFLUCC 23–0066 groups with the unclassified sequence of UM1559, and MFLUCC 23–0296 clusters with JEL0923, MP57, JEL0930 and WJD130. Our new *Terramyces* isolate MFLUCC 23–0298 groups with ARG040. Prior to this study, *Alphamyces* and *Pateramyces*, each contained only one species. Our phylogenies placed the new isolates in these genera, but clearly segregated them from the type sequences.

**Fig. 2:**
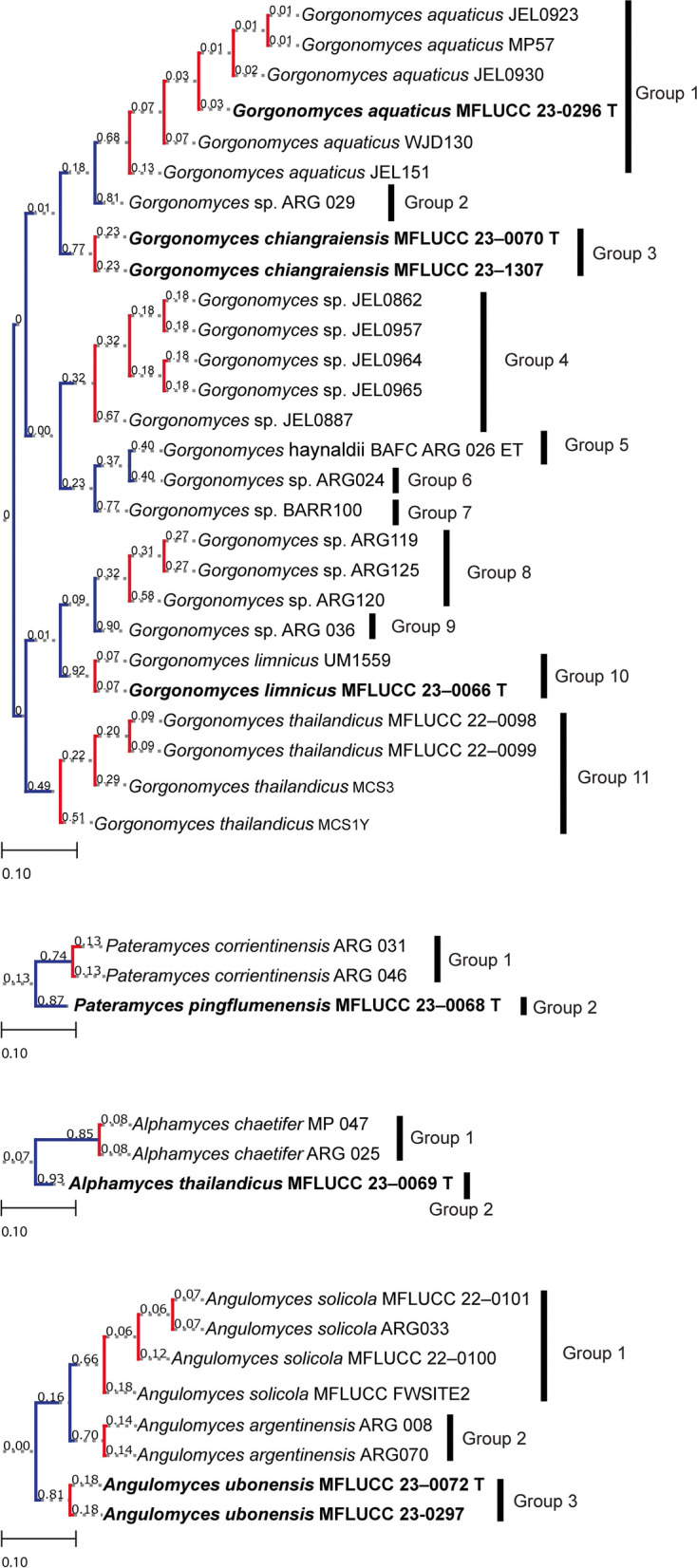
Results obtained from PTP analyses *Alphamyces*, *Angulomyces*, *Gorgonomyces*, *Pateramyces*, and *Terramyces*. The analysis was based on the ML topologies inferred using ITS-LSU sequence data. Species clusters are indicated with red-colored branches. Numbers near the nodes are posterior probabilities

The PTP analysis results (Fig. 2) agreed with those of the inferred phylogeny concerning the novelty of the strains and species delimitation. Specifically, the PTP analysis indicated three distinct species within *Angulomyces*, eleven species in *Gorgonomyces* and six species in *Terramyces*. The genetic distances of the trimmed dataset of the new taxa and sister taxa were measured (Tables 2, 3, 4, 5 and 6). *Angulomyces* was represented by three clades and the genetic distance between them was 2–7% (Table 3). *Gorgonomyces* isolates grouped into eleven clades with genetic distance between clades ranging from 1.5 to 10.5% (Table 5). Following the phylogenetic species concept, *Terramyces* was split into four clades each representing a species; the average genetic distance between species was 1.1–9.2% (Table 6). In both *Alphamyces* and *Pateramyces*, PTP results indicate two clades with a interspecies genetic distances of 20% (Fig. 2; Tables 2 and 4).

**Table 2:** Genetic distance (%) between *Alphamyces* strains (grouped according to PTP results) in the ITS genetic marker (603 bp)

	*Alphamyces chaetiferum*
***Alphamyces thailandicus***	20

**Table 3:** Genetic distance (%) between *Angulomyces* strains (grouped according to PTP results) in the ITS genetic marker (662 bp)

	*Angulomyces argentinensis*	*Angulomyces solicola*
*Angulomyces argentinensis*	0	
*Angulomyces solicola*	2	0
***Angulomyces ubonensis***	7	6.5

**Table 4:** Genetic distance (%) between *Pateramyces* strains (grouped according to PTP results) in the ITS genetic marker (647 bp)

	*Pateramyces corrientinensis*
***Pateramyces pingflumenensis***	20

**Table 5:** Genetic distance (%) between *Gorgonomyces* strains (grouped according to PTP results) in the ITS genetic marker (472 bp)

	**Group 1**	Group 2	**Group 3**	Group 4	Group 5	Group 6	Group 7	Group 8	Group 9	**Group 10**
Group 1										
Group 2	0.8									
**Group 3**	4.8	5.5								
Group 4	4.1	5.2	6.4							
Group 5	5.5	5.0	5.9	5.5						
Group 6	5.4	5.0	5.9	5.5	0.9					
Group 7	5.4	5.6	6.1	5.4	1.8	1.5				
Group 8	5.5	6.6	6.2	6.3	6.3	6.8	6.3			
Group 9	5.6	6.6	6.3	6.4	5.3	6.1	6.3			
**Group 10**	5.2	6.3	4.9	6.0	5.7	6.3	5.2	2.0	2.0	
Group 11	9.9	10.8	9.8	10.1	8.9	9.2	8.6	8.9	9.0	8.9

**Table 6:** Genetic distance (%) between *Terramyces* strains (grouped according to PTP results) in the ITS genetic marker (568 bp)

	Group 1	Group 2	Group 3	Group 4	Group 5
Group 1					
Group 2	4.7				
Group 3	5.2	1.3			
Group 4	5.3	1.1	2.4		
**Group 5**	7.7	5.9	7.0	7.4	
**Group 6**	9.2	7.6	9.0	8.1	4.4

## Taxonomy

***Alphamyces*** Letcher et al., *Mycol. Res.* 112 (7): 772 (2008)

MycoBank no.: MB 511785

*Generic description*: Sporangium spherical with a single discharge pore, the upper two thirds of the sporangial wall covered with long slender branched or unbranched hairs, sometimes slightly angular near the discharge papillae. Rhizoids branched. Zoospore contains a single, rather small lipid globule partially covered with a fenestrated cisterna. Mitochondrion single, a portion of which lies above and proximal to the kinetosome. Moderately electron-dense walled vesicles occur in the cytoplasm adjacent to the kinetosome. Based on Letcher et al. (2008).

*Type*: *Alphamyces chaetiferum* (Sparrow) Letcher 2008.

*Distribution:* Argentina, Thailand, and USA.

***Alphmyces thailandicus*** V.G Hurdeal & E. Gentekaki, **sp. nov.**

*MycoBank*: MB 848670

*Etymology*: Epithet references the country from where the species was isolated.

*Diagnosis*: *Alphamyces thailandicus* is characterized by significantly larger sporangia (27–59.5 µm diam.) than *A. chaetiferum* (15–22 µm diam.). 

**Fig. 3:**
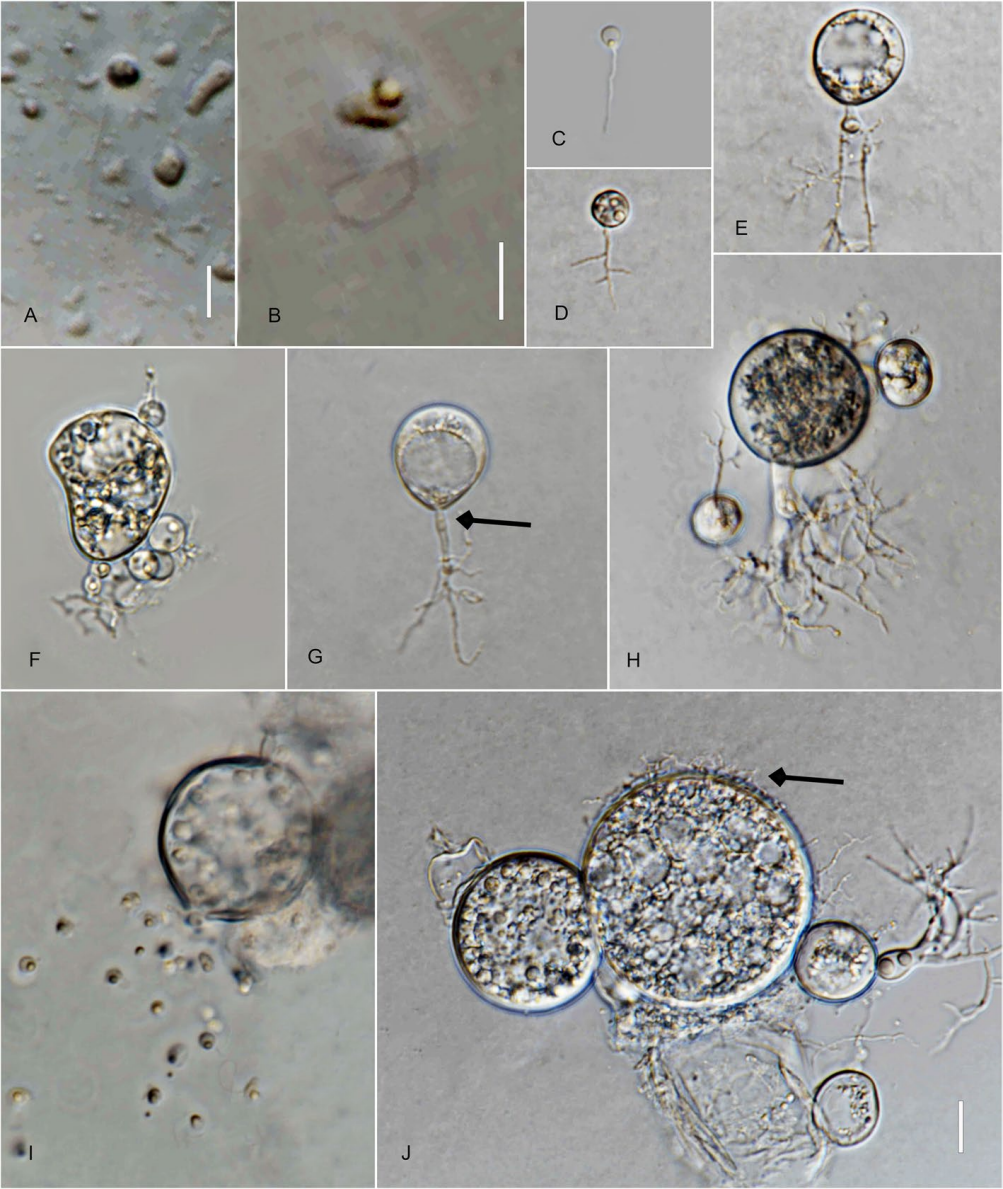
*Alphamyces thailandicus* (holotype) **A**, **B** zoospores; **C**, **D** developing germling; **E**–**H J** developing sporangium with one rhizoidal axis (arrow); **I** release of zoospores from sporangium; **J** sporangium with hair-like extensions (arrow). Bars: **A**, **B** = 5 µm; **C**–**J** = 10 µm

*Type*: **Thailand**: *Ubon Ratchathani Province*: Trakan Phuet Phon District, 15°32′48.0"N, 104°58′36.0"E, from water/sandy sediment samples baited with pollen, May 2022, *B. Raghoonundon* [isol*. *V.G. Hurdeal] (Fig. 3 in this paper – Holotype; MFLUCC 23–0069 – ex-type living culture).

*Description*: Light microscopy, on PmTG medium: thallus monocentric, eucarpic, epibiotic, sporangia spherical, 27–59.5 µm ($\overline {\text X}$ = 37 µm, *n* = 34), 1 short discharge papilla at maturity, with one rhizoidal axis. Immature thalli have single long branched rhizoids that gradually taper and become moderately (sometimes extensively) branched. Zoospores oval to spherical, 3–4 µm diam. ($\overline {\text X}$ = 3.5 µm, *n* = 35). Resting spores not observed. Generation time on PmTG at 20 °C 2–3 days.

*Notes*: Phylogeny indicates clear distinction of the new isolate from *A. chaetiferum* with high statistical support obtained from maximum likelihood (IQ-TREE, RAxML) and Bayesian inference. The genetic distance between the types of *A. chaetiferum* and *A. thailandicus* in the trimmed ITS region is 20%.

*Distribution*: Thailand.

***Angulomyces*** Letcher, *Mycol. Res.* 112(7): 776 (2008).

*MycoBank*: MB 511779.

*Generic description and notes*: See Hurdeal et al. (2023) and Letcher et al. (2008).

*Type species*: *Angulomyces argentinensis* Letcher et al. 2008.

Distribution: Argentina, Malaysia, Thailand, and USA.

***Angulomyces ubonensis*** V.G. Hurdeal & E. Gentekaki** sp. nov.**

*MycoBank*: MB 848669

*Etymology*: Epithet refers to the province from where the species was isolated.

*Diagnosis*: *Angulomyces ubonensis* differs both morphologically and phylogenetically from *A. argentinensis* and *A. solicola*. The newly described species produces smaller sporangia (to 29 µm *vs* 35 and 41 µm for *A. argentinensis* and *A. solicola*, respectively), with usually only one discharge papilla (numerous in *A. argentinensis* and to two in *A. solicola*). Zoospores vary slightly whereby they are smaller (2.5–4.5 µm diam.) than *A. solicola* (3–4 µm diam.) but larger than the average of *A. argentinensis* (5.5 µm diam.).

**Fig. 4:**
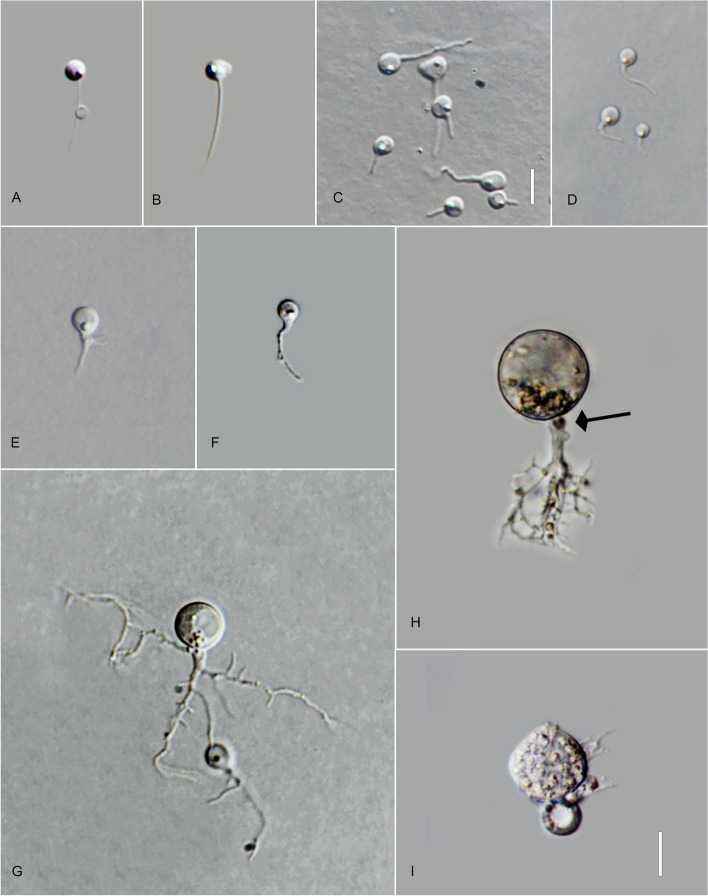
*Angulomyces ubonensis* (holotype) **A**, **B** zoospores; **C**-**F** developing germling; **F**–**H** developing sporangium with one rhizoidal axis (arrowed); **L** mature sporangia. Bar: **A**–**I** = 10 µm

*Type*: **Thailand**: *Ubon Ratchathani Province*: Khueang Nai District, 15°17′27.0″N, 104°38′42.0″E, from muddy river samples baited with pollen, May 2022, *B. Raghoonundon* [isol. by V.G. Hurdeal] (Fig. 4 in this paper – Holotype; MFLUCC 23–0072 – ex-type living culture).

*Description*: Light microscopy, on PmTG media: thallus monocentric, eucarpic, epibiotic, sporangia spherical, angular, 16–29 µm ($\overline {\text X}$ = 23.5 µm, *n* = 60), 1–2 short discharge papillae at maturity, but most frequently no discharge papillae are observed in this medium, usually with one rhizoidal axis (occasionally two), gradually tapering with branched rhizoids, often the single axis branches into 2 main sub branches and tapers into finer rhizoids. Rhizoids moderate to profusely branched. Zoospores oval to spherical, 2.5–4.5 µm diam. ($\overline {\text X}$ = 3 µm, *n* = 35), flagellum 7.5–15 µm ($\overline {\text X}$ = 11.5 µm, *n* = 30). Resting spores not observed. Generation time on mPmTG at 20 °C 2–3 days.

*Notes*: Phylogenetic analyses indicate three distinct species with maximum bootstrap support, and PTP specification. The pairwise nucleotide differences in the trimmed ITS (634 bp) of *A. ubonensis* to *A. argentinensis* and *A. solicola* are 7% and 6.5% respectively.

*Other material examined*:** Thailand**: *Ubon Ratchathani Province*: Khueang Nai District, 15°17′27.0"N, 104°38′42.0"E, from muddy river samples baited with pollen, May 2022, *B. Raghoonundon* [isol. by V.G. Hurdeal] (MFLUCC 23–0297).

Distribution: Thailand.

***Gorgonomyces*** Letcher, *Mycol. Res.* 112 (7): 767 (2008)

*MycoBank:* MB 511769.

*Generic description and notes*: See Hurdeal et al. (2023) and Letcher et al. (2008).

*Type species*: *Gorgonomyces haynaldii* (Schaarschm.) Letcher 2008.

*Distribution:* Argentina, Canada, South Korea, Thailand, and USA.

***Gorgonomyces aquaticus*** V.G. Hurdeal, & E. Gentekaki **sp. nov.**

*MycoBank*: MB 848671

*Etymology*: Epithet refers to the aquatic environment from where the species was isolated.

*Diagnosis:* Distinct from *G. haynaldii* (ARG 026 – epitype) by having smaller zoosporangial diameter (to 50 µm in *G. haynaldii*), fewer, shorter, and smaller discharge papillae (10–19 µm). Compared to *Gorgonomyces thailandicus*, *G. aquaticus* can produce longer discharge tubes and larger zoospores.

**Fig. 5:**
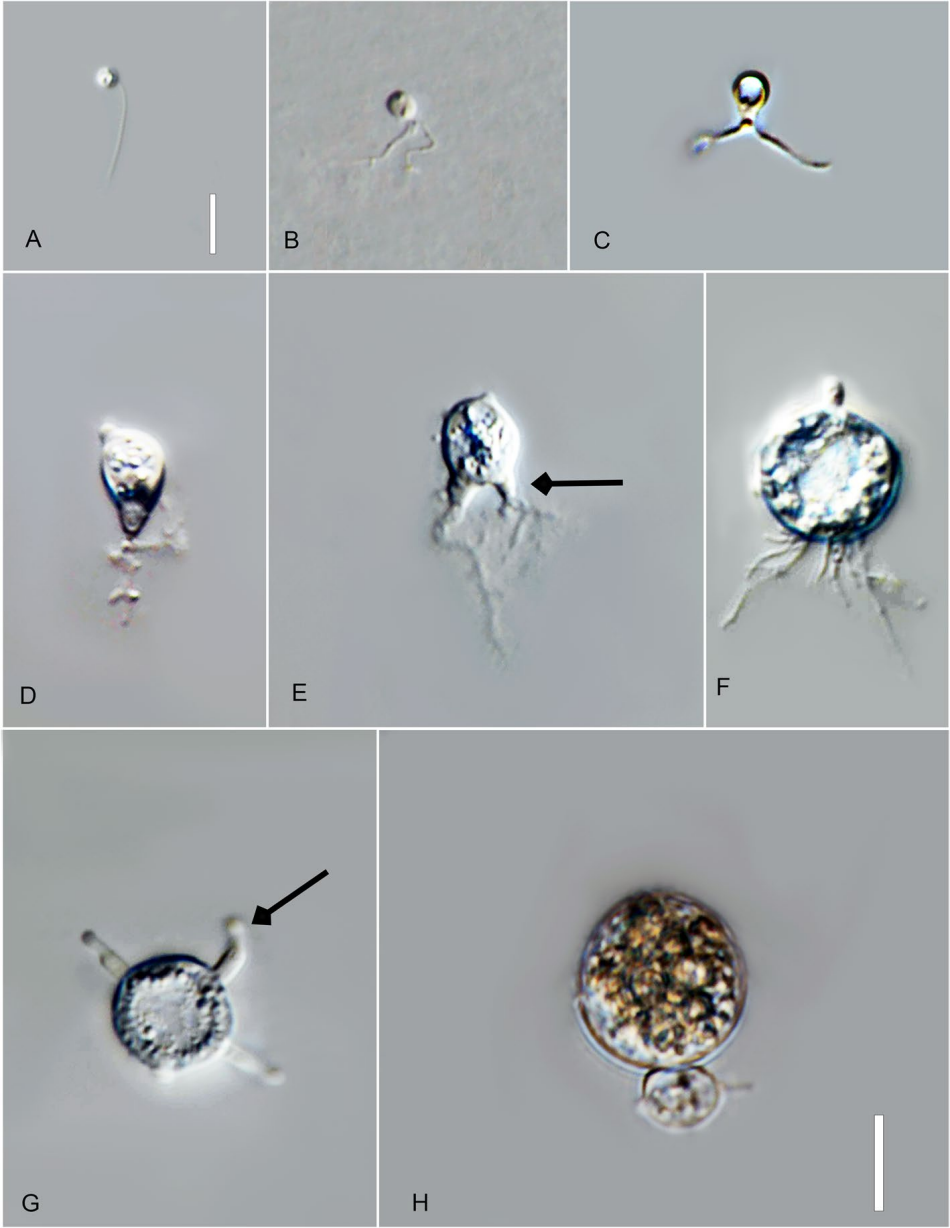
*Gorgonomyces aquaticus* (holotype) **A** zoospores; **B**, **C** developing germling; **D**–**H** developing sporangium with one or two rhizoidal axes; **E** developing sporangium with two rhizoidal axes (arrow); **G** sporangium with discharge papillae (arrow); **H** mature sporangium. Bar: **A**–**H** = 10 µm

*Type*: **Thailand**: *Chiang Rai Province*: Mai Sai District, from water baited with pine pollen, Jan. 2022, *V.G. Hurdeal* (Fig. 5 in this paper – Holotype; MFLUCC 23–0296 – ex-type living culture).

*Description*: Light microscopy, on PmTG medium: Thallus monocentric, eucarpic, epibiotic. Sporangia spherical, ovoid, clavate, 10–19 µm ($\overline {\text X}$ = 13.5 µm, *n* = 30) possessing 1 or more, long and moderate, undulate discharge papillae 3–8 × 3.5–8.5 µm ($\overline {\text X}$ = 5.5 µm long, *n* = 20) at maturity. Zoospore cyst produces mostly one to two rhizoidal axes. Rhizoids arise from cylindrical knob-like extension of the sporangium base. Zoospores oval to spherical, 3–4 µm diam ($\overline {\text X}$ = 3.5 µm, *n* = 20), posteriorly flagellated 10–14.5 µm ($\overline {\text X}$ = 11.5 µm, *n* = 20). Generation time on PmTG at 20 °C 1–2 days.

*Notes*: Phylogenetic analyses and genetic distances show that *G. aquaticus* clusters with another unclassified strain of *Gorgonomyces*, hence providing more resolution to the delineation of the new species and insights on the distribution of this species. The genetic distance of this new species to other described taxa ranges from 4.8–5.5%.

*Distribution*: Thailand, and USA.

***Gorgonomyces limnicus*** V.G. Hurdeal, & E. Gentekaki **sp. nov.**

*MycoBank*: MB 848672

*Etymology*: Epithet references the source (Greek λίμνη = lake) from where the species was isolated.

*Diagnosis*: *Gorgonomyces limnicus* is characterized by having smaller zoosporangia than *G. haynaldii* (ARG 026 – epitype) (to 50 µm in *G. haynaldii*), significantly fewer, shorter, and smaller discharge papillae but larger zoospores. This species differs from other *Gorgonomyces* strains introduced in this study and *G. thailandicus*, by having a different generation time, and larger sporangia.

**Fig. 6:**
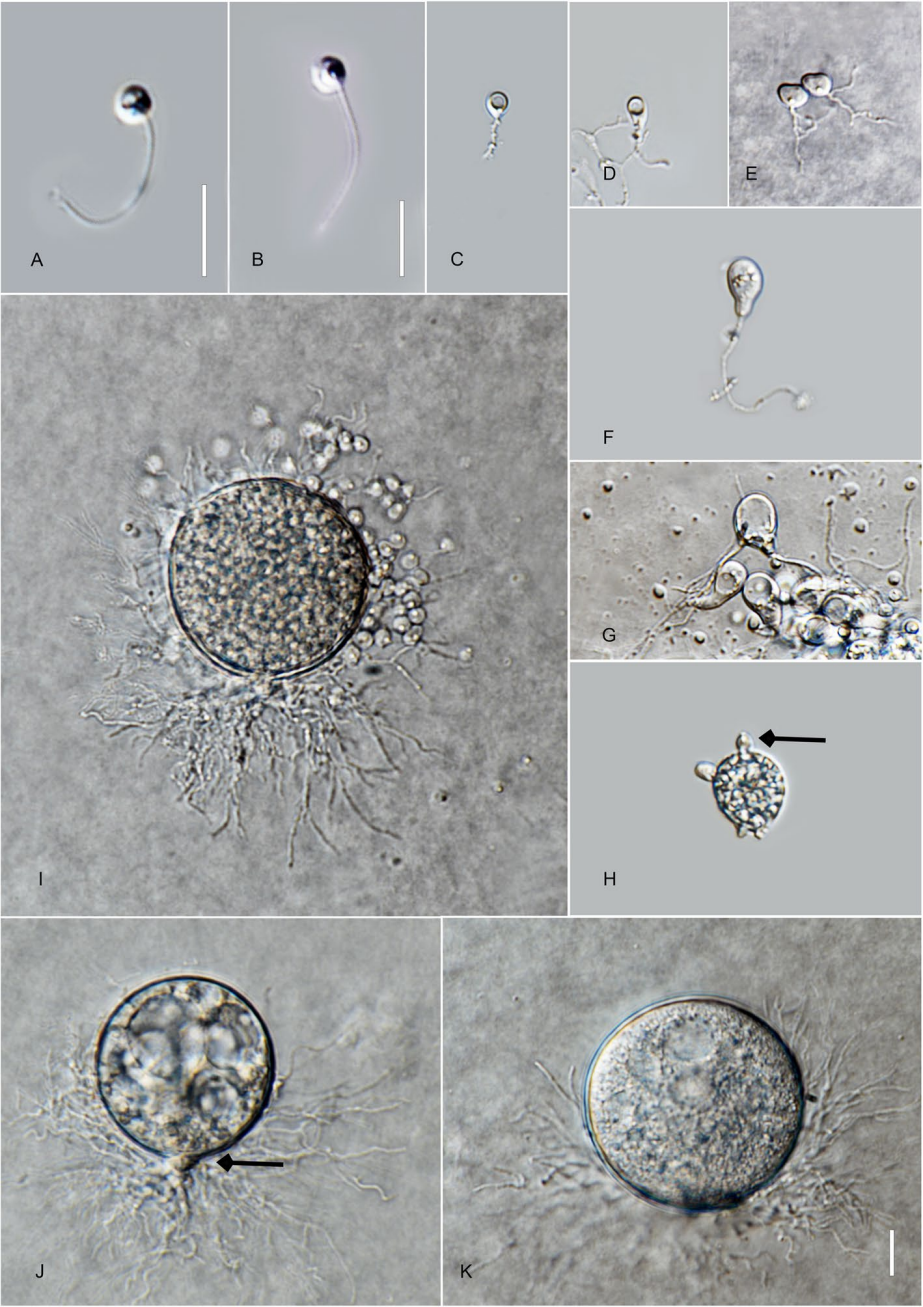
*Gorgonomyces limnicus* (holotype) **A**, **B** zoospores; **C**–**G** developing germling; **H**–**K** sporangium with knob-like rhizoidal axis (arrow); **H** sporangium with discharge papillae (arrow); **I**–**K** mature sporangium. Bars: **A**, **B** = 10 µm, **C**–**K** = 10 µm

*Type*: **Thailand**: *Chiang Rai Province*: Mai Sai District, from water baited with pine pollen, Jan. 2022, *V.G. Hurdeal* (Fig. 6 in this paper – Holotype; MFLUCC 23–0066 – ex-type living culture).

*Description*: Light microscopy, on PmTG medium: Thallus monocentric, eucarpic, epibiotic. Sporangia globose, 17–49 µm ($\overline {\text X}$ = 44.5 µm, *n* = 30) and possessing 1–4 short discharge papillae at maturity. The zoospore cyst produces mostly one to three rhizoidal axes. Rhizoids arise from cylindrical knob-like or slightly tubular extension of the sporangium base, extensively branched. Zoospores oval to spherical, 3.5–4.5 × 3.4–5 µm diam. ($\overline {\text X}$ = 4 µm, *n* = 20), posterior flagellum, 11–14.5 µm ($\overline {\text X}$ = 18 µm, *n* = 20). Generation time on mPmTG at 20 °C 3 days.

*Notes*: Phylogenetic analyses and genetic distances show that *Gorgonomyces aquaticus* is a new species. The distinct clading of the species to the other members is representative of a new species with a significant percentage pairwise difference in the ITS. The genetic distance of this new species to other described taxa ranges from 4.9–5.7%.

*Distribution*: Thailand, and USA

***Gorgonomyces chiangraiensis*** V.G. Hurdeal, & E. Gentekaki **sp. nov.**

*MycoBank*: MB 848674

*Etymology*: Epithet references the province from where the species was isolated.

*Diagnosis*: *Gorgonomyces chiangraiensis* has smaller sporangia and discharge papillae than *G. haynaldii* (ARG 026 – epitype) (to 50 µm in *G. haynaldii*). *Gorgonomyces chiangraiensis* produces more discharge papillae than *G. thailandicus* (to 4 in *G. thailandicus*), *G. limnicus* and *G. aquaticus*. This new species differs from other *Gorgonomyces* strains introduced in this study by having a different generation time, and larger sporangia.

**Fig. 7:**
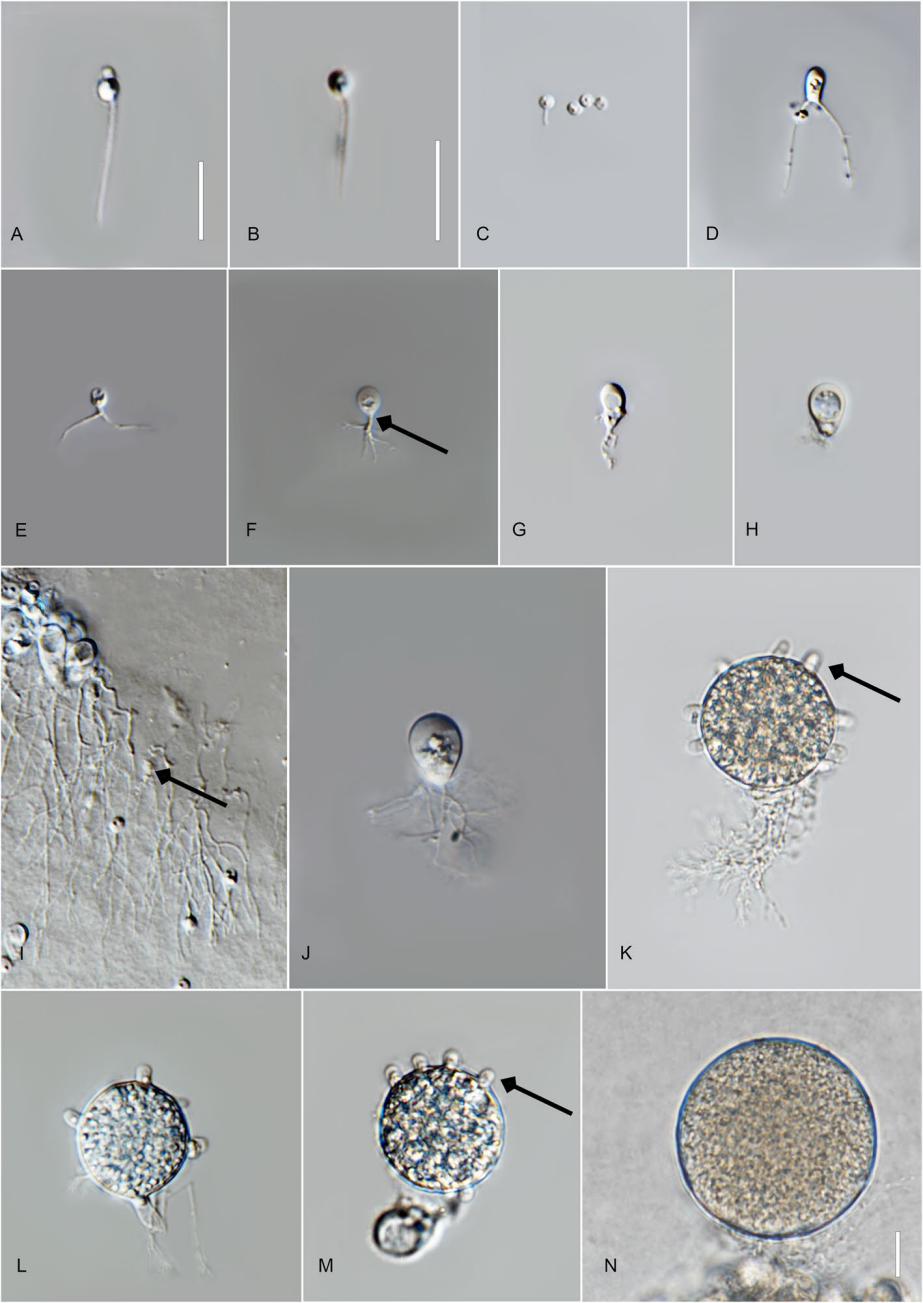
*Gorgonomyces chiangraiensis* (holotype) **A**, **B** zoospores; **C**–**I** developing germling; **F** rhizoidal axis (arrow); **I** germlings with extensive rhizoidal branching; **J–N** sporangia; **K–M** sporangia with discharge papillae. Bar: **A**–**K** = 10 µm

*Type*: **Thailand**: *Chiang Rai Province*: Mae Chan District, from water baited with pine pollen, Jan. 2022, *V.G. Hurdeal* (Fig. 7 in this paper – Holotype; MFLUCC 23–0070 – ex-type living culture).

*Description*: Light microscopy, on PmTG medium: Thallus monocentric, eucarpic, epibiotic. Sporangia spherical, 23–44.5 µm ($\overline {\text X}$ = 28 µm, *n* = 30) and possessing several short discharge papillae at maturity. Zoospore cysts produce mostly-one to two rhizoidal axes. Rhizoids arise from cylindrical knob-like extension of the sporangium base. Zoospores oval to spherical, 2–3 µm diam. ($\overline {\text X}$ = 2.5 µm, *n* = 20), posterior flagellum 8–15 µm ($\overline {\text X}$ = 13 µm, *n* = 20). Generation time on mPmTG at 20 °C 2 days.

*Notes*: Phylogenetic analyses and genetic distances also indicate the novelty of *G. chiangraiensis*. The genetic distance of this new species to other described taxa ranges from 4.8–6.4%.

*Other material examined*: **Thailand:** *Chiang Rai Province*: Mae Chan District, from lake water baited with pollen, Jan. 2022, *V.G. Hurdeal* (MFLUCC 23–1307).

*Distribution*: Thailand.

***Pateramyces*** Letcher, *Mycol. Res.* 112 (7): 779 (2008)

*Generic description*: Sporangium spherical with one discharge pore. Zoospores with one lipid globule partially covered with a fenestrated cisterna. Mitochondrion single. Based on Letcher et al. (2008).

*Type species*: *Pateramyces corrientinensis* Letcher 2008.

*Notes*: *Pateramyces* was introduced to accommodate three chytrids isolated from a water sample collected from a small lake in Argentina, and baited with pollen. Letcher et al. (2008), based the new genus on morpho-phylogenetic analyses. In the inferred ITS-LSU phylogenetic analysis, the taxon clustered sister to Rhizophydiaceae, leading to the introduction of a new family and genus. Morphological characterization indicated that *Pateramyces corrientinensis* isolates produced spherical sporangia at maturity, each with an operculate discharge tube. *Pateramyces pingflumenensis* possesses similar morphological characteristics. Genetic distance analyses (Table 4) in ITS further validated the introduction of the new species.

***Pateramyces pingflumenensis*** V.G. Hurdeal, & E. Gentekaki **sp. nov.**

*MycoBank*: MB 848668

*Etymology*: The species epithet refers to the Ping River (Latin flumen = river), from which the strain was isolated.

*Diagnosis*: *Pateramyces pingflumenensis* produces smaller sporangia (15 µm) than *P. corrientinensis* (to 30 µm) and slightly larger zoospores (5 µm vs 4 µm).

**Fig. 8:**
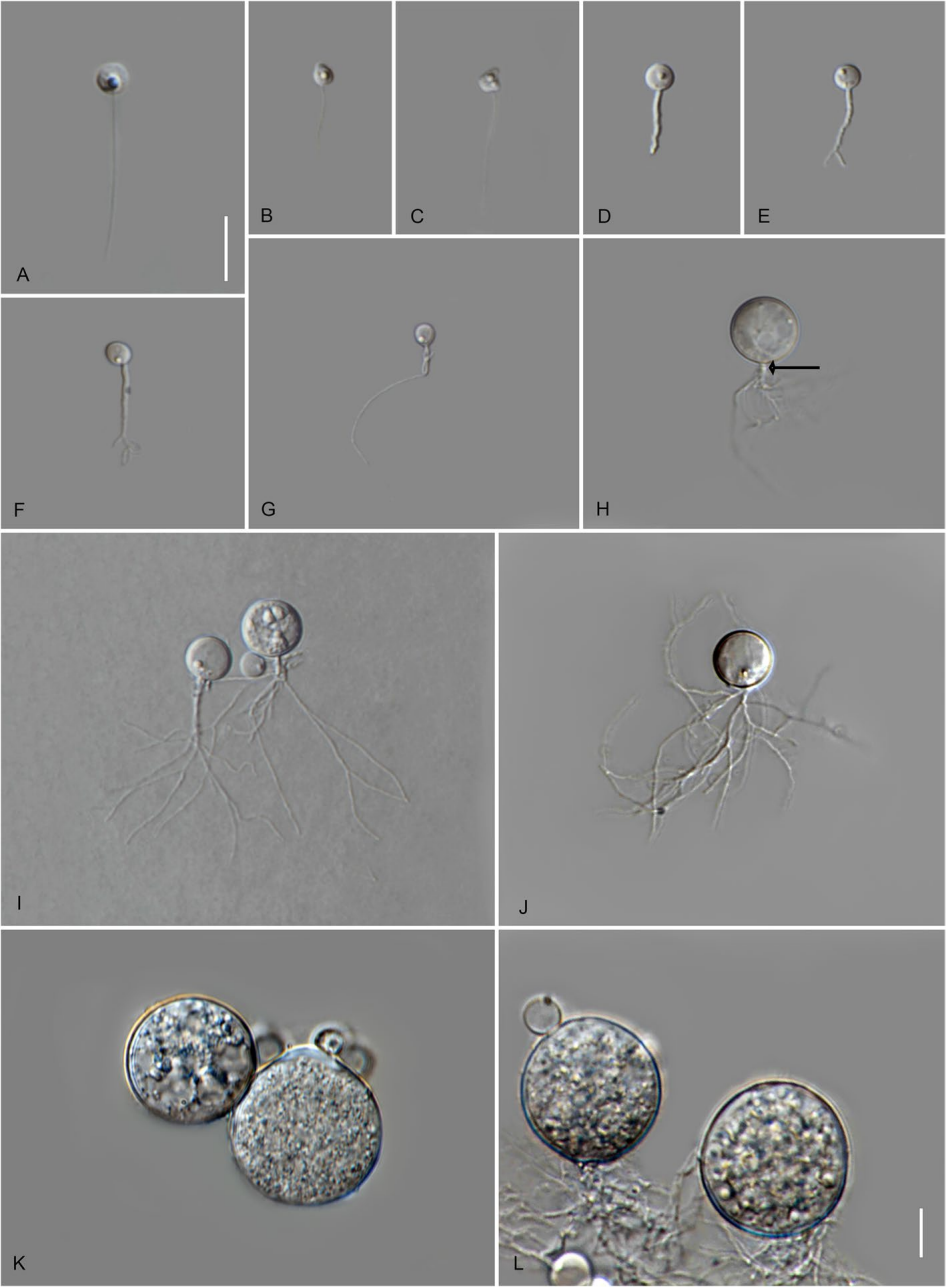
*Pateramyces pingflumenensis* (holotype) **A** zoospores; **B**–**G** developing germling; **H**–**L** developing sporangium with one rhizoidal axis; **L** mature sporangium. Bars: **A** = 10 µm, **B**–**K** = 10 µm

*Type*: **Thailand:** *Chiang Mai Province*: Mueang District, Ping River, 20° 1′ 14.2464″N, 99° 52′ 11.0742″E, from water samples baited with pollen, March 2022, *V.G. Hurdeal* (Fig. 8 in this paper – Holotype; MFLUCC 23–0068 – ex-type living culture).

*Description*: Light microscopy on PmTG medium: thallus monocentric, eucarpic, epibiotic; sporangia spherical at maturity, 12.5–18 µm ($\overline {\text X}$ = 15 µm, *n* = 30). Operculate sporangia were not observed. One rhizoidal axis with gradually tapering and branched rhizoids. Rhizoids moderate, sometimes profusely branched. Zoospores oval to spherical, frequently distorted in shape, 3.5–5 µm diam. ($\overline {\text X}$ = 4.5 µm, *n* = 30), flagellum 20–26 µm ($\overline {\text X}$ = 24.5 µm,* n* = 30). Resting spores not observed. Generation time on mPmTG at 20 °C 2 days.

*Notes*: The phylogeny indicates a clear distinction from *P. corrientinensis* with maximum statistical support obtained from maximum likelihood (IQ-TREE, RAxML) and Bayesian inference. The genetic distance between the type of *P. corrientinensis* and *P. pingflumenensis* in the trimmed ITS region is 20%.

*Distribution*: Thailand.

***Terramyces*** Letcher *Mycol. Res.* 110 (8): 911 (2006).

MycoBank: MB 29046.

*Generic description and notes*: See Hurdeal et al. (2023) and Letcher et al. (2008).

*Type species*: *Terramyces subangulosum* (A. Braun) Letcher 2006.

*Distribution:* Australia, Brazil, England, New Zealand, Thailand, and USA.

***Terramyces flumenensis*** V.G. Hurdeal, & E. Gentekaki **sp. nov.**

*MycoBank*: MB 848675

*Etymology*: Epithet refers to the environment from which the species was isolated.

Diagnosis: *Terramyces flumenensis* has notably larger sporangia than any other currently described *Terramyces* species.

**Fig. 9:**
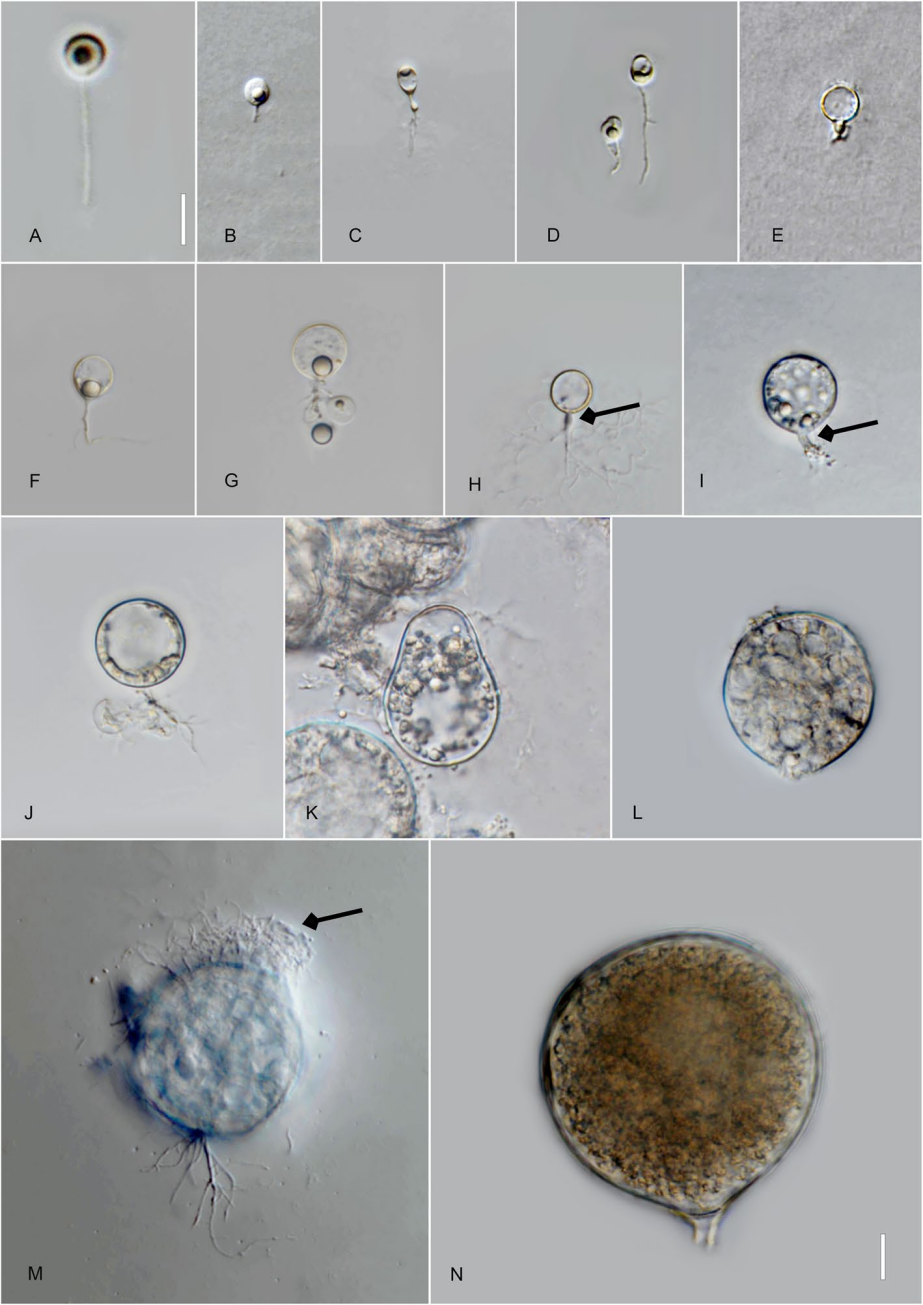
*Terramyces flumenensis* (holotype) **A** zoospores; **B**-F developing germling; **G**-**N** developing sporangium with one rhizoidal axis; **M** sporangium with hair-like structures; **N** mature sporangium. Bars: **A** = 5 µm; **B**–**N** = 10 µm

*Type*: **Thailand**: *Ubon Ratchathani Province*: Khueang Nai District, 15°17′27.0″N, 104°38′42.0″E, from muddy river water baited with pollen, May 2022, *B. Raghoonundon* [isol. by V.G. Hurdeal] (Fig. 9 in this paper – Holotype; MFLUCC 23–0067 – ex-type living culture).

*Description*: Light microscopy, on PmTG medium: thallus monocentric, eucarpic, epibiotic, sporangia spherical, angular, sometimes irregular in shape, 24.5–80 µm ($\overline {\text X}$ = 58 µm, *n* = 30) with fine hair-like structures on the surface. 1–2 short discharge papillae, but frequently no discharge papillae are observed on this medium, usually with one knob-like to tubular rhizoidal axis (occasionally two). Rhizoids moderately to profusely branched. Zoospores oval to spherical, 4–4.5 µm diam. ($\overline {\text X}$ = 4.5 µm, *n* = 20). Resting spores not observed. Generation time on mPmTG at 20 °C 3 days.

*Notes*: Genetic analysis shows that this new species is clearly different from the type species in the genus. The genetic difference between this new species and others in the same genus is between 4.4% and 9.2%. PTP analysis confirms that this is indeed a new and distinct species.

*Other material examined*: **Thailand:** *Chiang Rai Province*: Mae Chan District, lake water, Jan. 2022, *V.G. Hurdeal* (MFLUCC 23–0071).

*Distribution*: Thailand.

***Terramyces aquatica***V.G. Hurdeal, & E. Gentekaki **sp. nov.**

*MycoBank*: MB 848676

*Etymology:* Epithet refers to the environment from where the species was isolated.

Diagnosis: This newly identified species differs from *T. subangulosum* (specifically the ARG-033 – epitype) by having larger sporangia (to 67 µm diam.), however, it has smaller sporangia and zoospores compared to *T. flumenensis*.

**Fig. 10:**
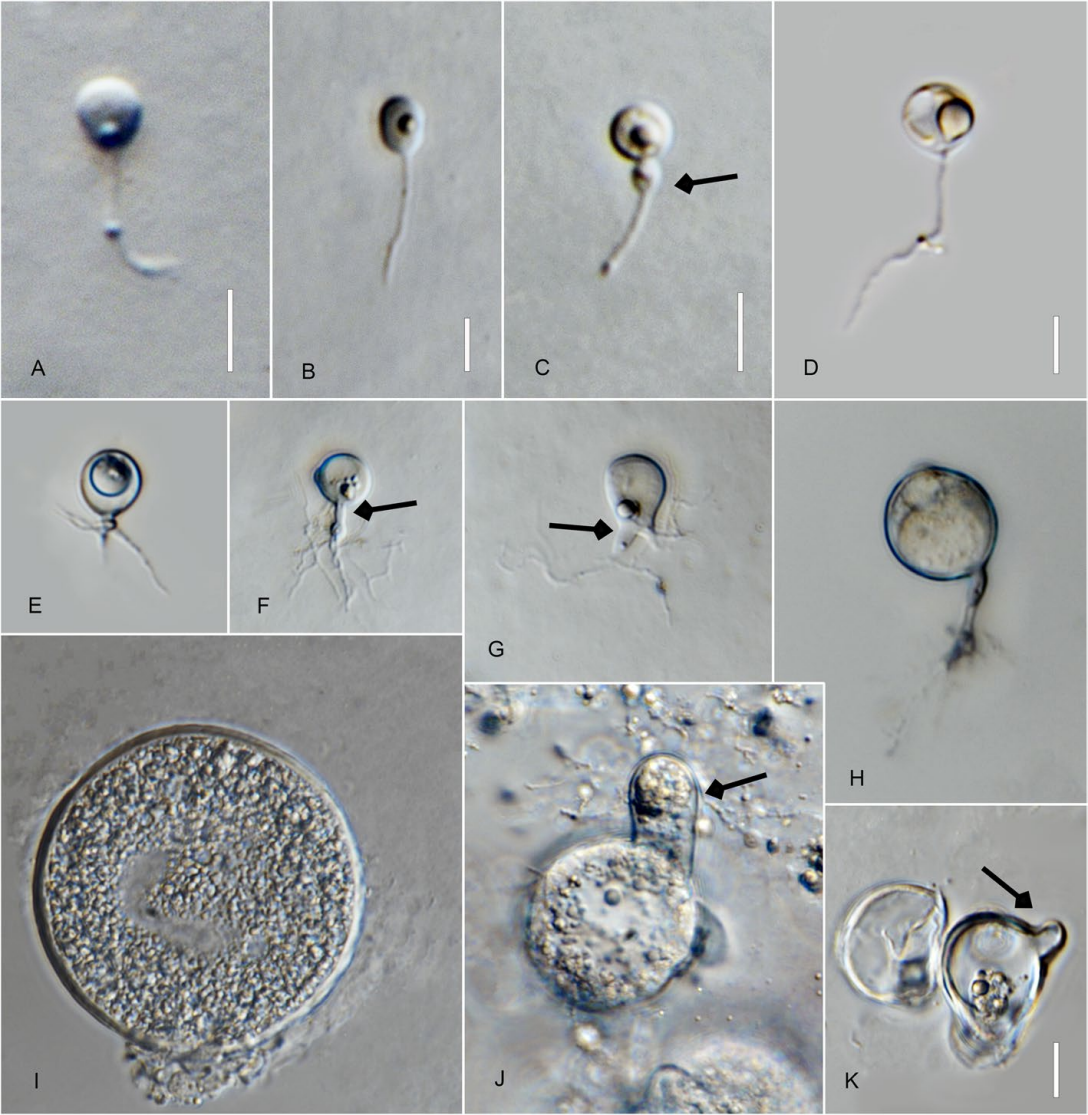
*Terramyces aquatica* (holotype) **A** zoospore; **B**–**D** developing germling; **E**, **F**, **H** developing sporangium with one rhizoidal axis (arrow); **G** developing sporangium with two rhizoidal axes (arrow); **I** mature sporangium; **J**–**K** sporangium with one discharge tube (arrow). Bars: **A**–**D** = 5 µm; **E**–**l** = 10 µm

*Type*: **Thailand:** *Chiang Rai Province*: Mae Chan District, from lake water baited with pollen, May 2022, *V.G. Hurdeal* (Fig. 10 in this paper – Holotype; MFLUCC 23–0298 –ex-type living culture).

*Description:* Light microscopy, on PmTG media: Thallus monocentric, eucarpic, epibiotic. Sporangia spherical becoming slightly angular at maturity. Sporangia 26.5–67 µm ($\overline {\text X}$ = 45.5 µm, *n* = 30). Thallus comprises one rhizoidal axis with extensive, branched rhizoids. Zoospores 4–4.5 µm diam. ($\overline {\text X}$ = 4.5 µm, *n* = 20). Generation time on mPmTG at 20 °C is 3–4 days.

*Notes*: Genetic analyses reveal that this new species forms a separate branch distinct from the type species and other described species in the genus. The genetic distance in the trimmed ITS between the novel species and others in the same genus ranges from 4.4% to 7.7%. Additionally, PTP analysis confirms the uniqueness of this newly discovered species.

*Distribution*: Thailand, and USA.

## Discussion

Chytrid taxonomy has experienced significant changes over the years. Early taxonomy was based on the morphological species concept (e.g., Sparrow 1943, 1960; Karling 1977). In the 1980s, the features used for the identification of chytrids changed drastically with the implementation of zoospore ultrastructure based on transmission electron microscopy (Barr 1980). Currently, the gold standard for the establishment of new taxa incorporates both morphological and molecular data. Species delimitation can bypass ultrastructure data because resolution provided by TEM is not definitive at the species level (Hurdeal et al. 2023). Furthermore, obtaining good quality ultrastructure data is often a bottleneck as not all species produce zoospores abundantly. Also, the expertise of interpreting zoospore ultrastructure data is limited, and the equipment not widely accessible to researchers. Hence, this slows down progress in describing the largely uncharacterized chytrid diversity (Tables 5 and 6).

Diversity and distribution information for chytrids is significantly lower relative to that for members of the *Dikarya*. However, steady progress has been made in the last twenty years, with many studies from the Americas (Letcher et al. 2008; Longcore 2004, 2011; Simmons et al. 2009, 2020, 2021; Wakefield et al. 2010; Marano et al. 2011; Longcore and Simmons 2012; Longcore et al. 2012; Vélez et al. 2013; Davis et al. 2015). Descriptions of new taxa are also coming from other parts of the globe (Seto and Degawa 2018a, b; van den Wyngaert et al. 2018; Hyde et al. 2019; Seto et al. 2020a, b; Karpov et al. 2021; Hurdeal et al. 2023). Collectively, these studies depict a broad distribution of chytrids in various parts of the world.

In this study, we increase the global knowledge of chytrids by introducing eight new rhizophydialean species from Thailand. Delineation of these species is based on a tripartite approach including morphological characterization, phylogenetic analyses based on ITS-LSU genetic markers and PTP. The morphology of the new species differs somewhat from the described species within the genera. Differences include the sizes of the reproductive structures and other morphological characters such as number of discharge papillae. However, because morphological characters are few and differ only slightly, we place the most emphasis on phylogenetic analyses of molecular data.

Phylogeny provided high statistical support for the establishment of most of the new taxa except in *Terramyces*, for which statistical support is low. This may be indicative of low resolution of the ingroups, or problematic sequences. Hence, in total in this study, the PTP analysis divided the currently known *Terramyces* strains into six groups, each group representative of a separate species. This reflects the effects of taxon sampling and the relative genetic distance among and within species. In all analyses, however, the placement of strains and taxa was stable. In *Terramyces*, the observed pairwise nucleotide differences spanned from 1.1% to 9%. The 1.1% divergence appears relatively low for the delineation of a new species. PTP suggests the possibility that groups 1–3 may indeed constitute a single taxonomic entity. This underscores the importance of employing the PTP method repeatedly as new strains and species are uncovered. Hence, the iterative application of PTP is crucial for achieving a more stable and accurate taxonomic classification.

Similarly, in *Gorgonomyces* the type strain *Gorgonomyces haynaldii* ARG 026 segregated from ARG 024, whereas previously the two grouped together. Hence, as Hurdeal et al. (2023) suggested, phylogenetic analysis is a dynamic process and as new strains become available, analyses are needed to validate the use of PTP or any new phylogenetic tools. Our results indicate that not only is generic diversity high but diversity is also high at the species level.

For the rest of the concerned monospecific genera, the PTP provided indication and evidence for the novelty of our isolates. However, as more strains and species become available, the analyses may need to be re-evaluated. Our new *Pateramyces* isolate differs by 20% from the *P. corrientinensis* clade in the trimmed ITS genetic marker indicating a high degree of genetic diversity in the genus.

*Pateramyces* and *Alphamyces* were previously monotypic genera isolated from Argentina. Letcher et al. (2008), introduced family and genus based on three isolates. From currently known data, the two genera are saprobes on pollen grains, whereas their diversity and distribution seem to be restricted to aquatic environments. Hence, it is evident from this and previous studies, that our knowledge of chytrid diversity remains quite limited. Consequently, the precision of the methods employed and their morphological characteristics are still uncertain. Future research will require thorough investigation into the ecology, re-evaluation of various taxonomic ranks, and the study of historical morphological species in order to gain a comprehensive understanding of this group of fungi.

## Data Availability

The final concatenated matrix and ML trees were deposited to Figshare (10.6084/m9.figshare.24910779). The sequence data generated in this study will be available in NCBI upon publication.
