# Correction: Diversity of *Rhizophydiales* (*Chytridiomycota*) in Thailand: unveiling the hidden gems of the Kingdom

**DOI:** 10.1186/s43008-024-00173-6

**Published:** 2024-12-16

**Authors:** Vedprakash G. Hurdeal, Joyce E. Longcore, E. B. Gareth Jones, Kevin D. Hyde, Eleni Gentekaki

**Affiliations:** 1 School of Science, Mae Fah Luang University, Chiang Rai, 57100 Thailand; 2 Center of Excellence in Fungal Research, Mae Fah Luang University, Chiang Rai, 57100 Thailand; 3 School of Biology and Ecology, University of Maine, Orono, ME 04469-5722 USA; 4 Department of Botany and Microbiology, College of Science, King Saud University, P.O. Box 2455, 11451 Riyadh, Saudi Arabia; 5 Department of Veterinary Medicine, University of Nicosia School of Veterinary Medicine, 2414 Nicosia, Cyprus

**Correction: IMA Fungus (2024) 15:17** 10.1186/s43008-024-00144-x

Following publication of the original article (Hurdeal et al. 2024), we noticed that the results of the PTP (Poisson Tree Processes) analyses for *Terramyces* were inadvertently omitted in Fig. 2. Below, we provide the missing image displaying the PTP analyses for *Terramyces*.


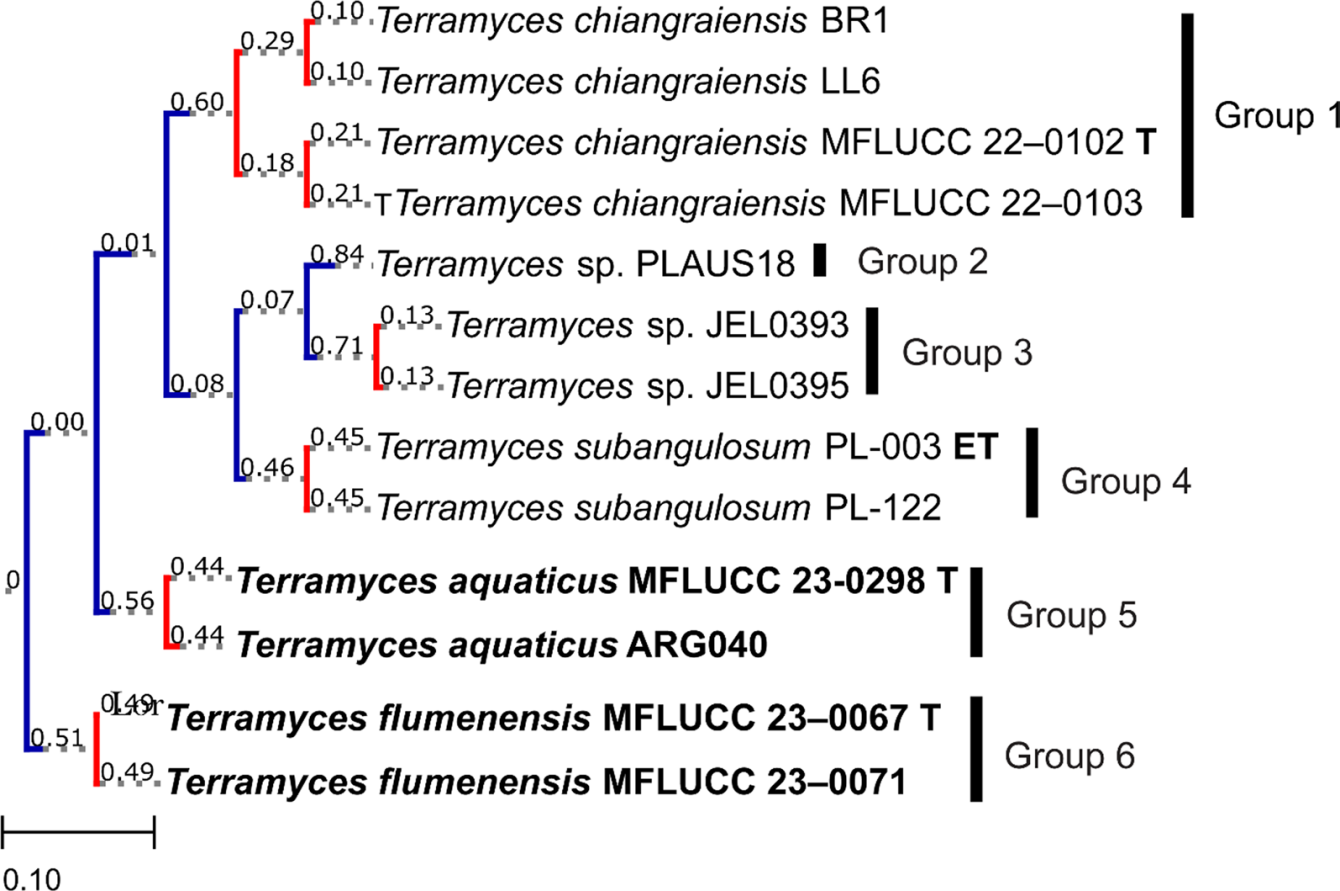


In the Taxonomy section of the published manuscript, there were two errors that require correction:

1. The species name “***Alphmyces thailandicus***” was misspelled. The correct name is ***Alphamyces thailandicus V.G. Hurdeal & E. Gentekaki, sp. nov.***
2. In the masculine genus ***Terramyces***, all adjectival species epithets must follow the masculine form. Therefore, the correct species name is ***Terramyces aquaticus V.G. Hurdeal & E. Gentekaki, sp. nov.*** rather than the previously published ***Terramyces aquatica***.
